# Outcome of Non-Hodgkin’s Lymphoma in Adults-Real World Experience from a Cancer Centre in North-East India

**DOI:** 10.1007/s12288-025-02097-6

**Published:** 2025-07-28

**Authors:** M Vinodhini, Roy Partha Sarathi, Iqbal Asif, Samba Siva Rao Pasupuleti, Hazarika Munlima

**Affiliations:** 1 Dept. of Medical Oncology/Haematology, All India Institute of Medical Sciences (AIIMS) Bibinagar, Hyderabad, India; 2 Dept. of Medical Oncology/Hematology, Dr. Bhubaneshwar Borooah Cancer Institute, (A Unit of Tata Memorial Hospital), Guwahati, India; 3 Dept. of Biostatistics, Mizoram Central University, Aizawl, India

**Keywords:** Non-Hodgkin’s lymphoma, Diffuse large B cell Lymphoma, Outcome, International prognostic index

## Abstract

**Background and Objectives:**

Non-Hodgkin’s lymphoma (NHL) represent diverse subtypes categorized as per immunohistochemical, genetic, molecular and clinical diagnosis delineated by 2016 World Health Organization classification. Given limited real-world data on NHL in North-East India, the objective was to investigate the response and outcome in this cohort.

**Materials and methods:**

Demographic, clinical, treatment and response characteristics of 214 NHL patients following frontline immunochemotherapy treated between December 2017 and January 2023 were retrospectively reviewed and analyzed to determine overall survival (OS) and event-free survival (EFS).

**Results:**

B cell-NHL accounted for 187 cases, followed by 27 T-NHL. Diffuse large B cell lymphoma (DLBCL) and peripheral T cell lymphoma, not otherwise specified, were the commonest B-NHL and T-NHL, respectively. Bulky disease was seen in 26(12.1%), advanced-stage in 137(64.01%), extra-nodal presentation in 77(35.98%),and grade 2 chemotherapy-related toxicity in 116(54.2%).The overall response rate was 73.36%, with complete response at 63.2% and progression at 20%. Median follow-up was 23 months (interquartile range 15–35 months). The 2-year EFS among those with complete/partial response was 77.7% compared to 24.53% among stable/progression. The 5-year OS was 47.84%. Those with good /low risk international prognostic index (IPI) had a 5-year OS of 62.09% compared to 25.17% among high/poor risk IPI. Most events, *n* = 72 out of 108 (66.6%), happened within 6–24 months of treatment completion.

**Conclusion:**

Despite treatment barriers, overall response and outcome were non-inferior and comparable to Western data. High-grade DLBCL, T-NHL, advanced stage, poor IPI attributed to suboptimal response and inferior survival, thus emphasizing novel prognostic markers and biological agents based on high-risk molecular signature to enable individualized treatment.

## Introduction

Non-Hodgkin’s lymphoma (NHL) is a common haematological malignancy. GLOBOCAN 2022 reported a crude cancer incidence rate of 100.4 per 100,000, with 105.4 per 100,000 among females and 95.6 per 100,000 among males, and cumulative risk of NHL in India as 1 in 284, with an age-adjusted rate of 3.8% in males and 2.5% in females. Worldwide, the age-standardized mortality rate for NHL was 1.9% among females and 2.9% among males [1].Although North America, Europe and Australia report higher incidence [1], changing lifestyle patterns, dietary habits, shift from rural to urban metropolitan cities, and demographic transition to older age, a more significant proportion of young Indians account for rising incidence and heterogeneity. As per the National Cancer Registry Programme, a population-based cancer registry from North-East India showed the highest incidence of cancers in both genders all over India.One in every four males belonging to Aizawl, Papumpare, East Khasi hills, Kamrup urban districts of North-East India and one in every four females of Mizoram state of North-East India are likely to develop cancer between 0 and 74 years age group. Due to a lack of infrastructure, human resources, access to specialized treatment centres, cultural factors and lifestyle choices, a substantially lower survival rate was reported in North-East India than in the rest of the country [2].

The paradigm shift from a homogenous management strategy to individualized risk categorization based on histopathology, immunohistochemistry, molecular markers and nuclear imaging-based response assessment enhanced response and survival in NHL over the last two decades. Evolving immunotherapies with checkpoint inhibitors, bispecific antibodies, and engineered T-cells are employed in current management worldwide.

The primary concern in the Indian setting is young age but advanced stage at presentation, poor performance status or comorbidities at diagnosis, incomplete adherence and impaired tolerance to standard treatment, causing a decline in survival rate compared to Western data [3].Given geographic variations, demographic and socio-economic influences, changing risk factors, rising incidence and chemo-resistance, the objective was to analyze the spectrum, response, and outcome of NHL patients treated in tertiary referral oncology centres in North-East India given limited available data.

## Materials and methods

This was a retrospective observational single institutional study conducted in our institute in patients with NHL over five years from August 2017 to January 2023. We evaluated electronic medical records as well as case files of patients ≥ 18 years of age with NHL, and data were collected for clinical history and presentation (age, performance status, B-symptoms, nodal or extra-nodal disease, organomegaly, superior vena cava syndrome, tumour lysis syndrome, malignant effusion or ascites, neurological manifestation, bone marrow involvement and bulky disease). Bulky disease was defined as mediastinal mass more than one-third of maximum transthoracic diameter or lymph node mass more than 7.5 cm in largest diameter. Extra-nodal disease was defined as contiguous involvement of non-lymphoid organs and primary extra-nodal as predominant involvement of non-lymphoid organs as major or initial disease site with no or only regional lymph node involvement. Eastern Oncology Co-operative Group (ECOG) performance status was captured at diagnosis and subsequent visit.

### Staging

Laboratory parameters such as complete haemogram, liver and renal function tests, blood glucose, serum electrolytes, uric acid, lactate dehydrogenase (LDH), beta-2-microglobulin, viral markers such as hepatitis B, hepatitis C and HIV, cerebrospinal fluid cytology, radiological classification as per Ann-Arbor staging with Cotswald’s modification using whole body fluorodeoxyglucose (FDG) avid positron emission tomography (PET-CT), computed tomography (CT) or magnetic resonance imaging (MRI) (when PET-CT was not feasible and in non-FDG avid histologies), tissue biopsy confirmation by 2016 World Health Organization (WHO) classification of NHL based on histopathology, immunohistochemistry (IHC) and break-apart fluorescence in-situ hybridization (FISH) were collected [4–7].Stage I and II were classified as limited stages, and stages III and IV were classified as advanced diseases. Stage IV included extensive involvement of lymphoid sites, bone marrow, liver and brain. Radiologically identifiable splenic mass or direct splenic infiltration was considered splenic involvement.

### Management

All patients fulfilling the diagnosis of NHL were treated as per standard protocol and recommended guidelines containing conventional chemotherapy or chemo-immunotherapy with CHOP-R (Cyclophosphamide, doxorubicin, vincristine, prednisolone, rituximab), EPOCH-R (etoposide, cyclophosphamide, doxorubicin, vincristine, prednisolone, rituximab), CHOEP (cyclophosphamide, doxorubicin, vincristine, etoposide, prednisolone), B-R(bendamustine, rituximab), MVP (methotrexate, vincristine, procarbazine), etoposide(E), ifosfamide (I), cytarabine (Ara-C), temozolomide, for four to six cycles in 21–28 days (as per protocol) and dose modified as per National Cancer Institute Common Terminology Criteria for Adverse events (NCI CTCAE version 5.0) haematological and organ toxicities in subsequent cycles [8].In selected histologies, ibrutinib, acalbrutinib, lenalidomide and venetoclax were preferred in second-line or chemo-refractory disease. Patients with bulky disease received 4–6 cycles of chemotherapy followed by involved field radiotherapy (IFRT). CNS prophylaxis with intrathecal methotrexate was administered if CNS-IPI *≥* 4 or if the involvement of the testis, adrenal, breast, or epidural space. Complete blood count, liver function test, and renal function test were undertaken before each cycle, and chemotherapy was administered if absolute neutrophil count was above 1000/mm^3^and less than grade 2 hepatic and renal toxicity. Granulocyte colony-stimulating factor (G-CSF) prophylaxis was administered when age *≥* 60 or when there is grade 3 to 4 neutropenia or neutropenic sepsis during chemotherapy.

### Response Evaluation

Assessment by PET-CT using a Lugano 5-point scale or CT/MRI scan was done four weeks after treatment completion. Complete response (CR), partial response (PR), and stable or progressive disease were assigned as per immune-related response criteria [9]. Patients were followed up and assessed clinically for any relapse every 3 months after treatment completion for the first 2 years and, after that, every 6 months for the next 3 years. When clinically suspected relapse, imaging and biopsy were repeated. The primary outcome was response rate and overall survival (OS). The secondary outcome was event-free survival (EFS) and chemotherapy-related toxicity. Overall survival was calculated from the date of diagnosis to the date of death due to any cause (censored when patients were lost to follow-up). The event was defined as either death, relapse or progression during follow-up after first-line treatment. The institutional ethics committee approved the study (project number: 13000506/dated 08/02/2023).

### Statistical Analysis

Descriptive statistics were presented as mean$\:\pm\:$standard deviation or median with interquartile range (IQR) for continuous variables and frequencies with percentages for categorical variables. The Chi-square test or Fisher’s exact test assessed bivariate associations between categorical variables. Either independent samples t-test or Mann-Whitney U-test was used to test the difference in mean values. The Cox proportional-hazards model expressed in the hazard ratio (HR) with confidence interval (CI) to identify factors associated with event-free and overall survival was estimated. Kaplan-Meier curves were used to depict the survival pattern, and the Log-rank test was used to assess the difference in survival. Data was censored in October 2024 for survival analysis. All statistical analyses were performed using STATA version 18 and SAS on Demand for Academics software. For statistical significance, a p-value less than 0.05 was considered.

## Results

**Fig. 1:**
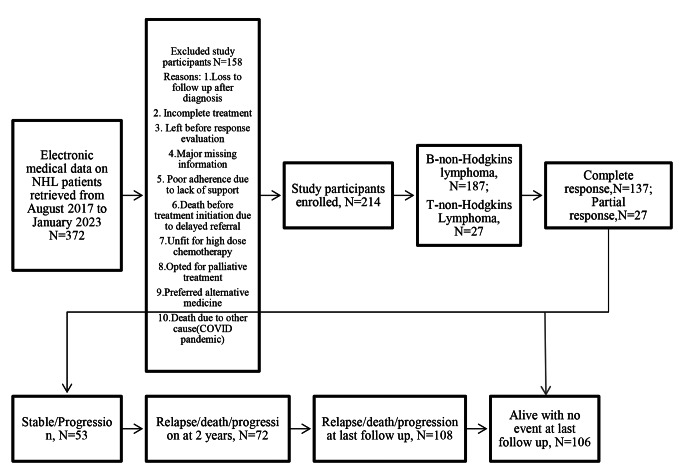
Consort diagram depicting the study of patient recruitment and attrition

There were a total of 214 NHL study cohorts. Figure 1 demonstrates the recruitment and attrition of study participants. Cervical lymphadenopathy was the most typical presentation, followed by fatigue, fever, and B-symptoms. Bulky disease was seen in 26 out of 214 cases (12.1%). B cell-NHL accounted for 187 cases, followed by 27 cases of T-NHL (excluding B and T- T-lymphoblastic lymphoma, which was not analyzed in the present study due to the same spectrum of acute lymphoblastic leukaemia, distinct driver mutations and similar response).

Diffuse large B cell lymphoma (DLBCL) was the commonest B-NHL(*n* = 121) followed by follicular, marginal and mantle zone lymphoma. Among T-NHL, peripheral T cell lymphoma, not otherwise specified (PTCL-NOS) was the commonest. There were 8 cases of primary CNS lymphoma (PCNSL), four plasmablastic lymphomas, primary mediastinal large B-cell lymphoma (PMBCL) and Waldenstrom’s macroglobulinemia (WM) each and two were Burkitt’s lymphoma and angio-immunoblastic T-cell lymphoma (AITL) each, one case of T-cell rich B-cell lymphoma (TCRBCL) and hepato-splenic gamma-delta T-cell lymphoma each. Table 1 illustrates the clinical, demographic and treatment characteristics. The advanced-stage disease was noted in 137 patients (64.01%). All patients with ALCL presented with stage IV disease (5/5 cases). The extra-nodal presentation was seen in 35.98% (77 cases), the most common site being the head and neck (37/77 cases), followed by the gastrointestinal tract (17/77 cases). Autoimmune manifestations such as skin rash, arthralgia, and hypergammaglobulinemia were seen in a few T-NHL. Revised international prognostic index (R-IPI), follicular lymphoma international prognostic index (FLIPI), mantle cell lymphoma international prognostic index (MIPI) tools prognosticated DLBCL, FL and MCL, respectively. Of 121 DLBCL including high grade (double/triple expressor or double/triple hit) 47.1% were identified with poor risk R-IPI, 40.74% had poor risk FLIPI and 25% with high risk MIPI scores.

**Table 1:** Demographic and disease characteristics

Median age (range) in years		54 (19–86)					
Male (range), in years		55 (19–86)					
Female (range), in years		52 (22–82)					
**Gender**							
Male: female (ratio)		159: 55 (2.8:1)					
**ECOG performance status**							
0–1 2–3 Not available		32 119 63					
**Serum Albumin**							
Less than 3.5 g/gl More than 3.5 g/dl Not available		22 98 94					
**Serum LDH**							
Normal Elevated Not available		112 45 57					
**Haemoglobin**							
Less than 10 g/dl More than 10 g/dl Missing		22 113 79					
**Extra-nodal Site (** *n* ** = 77)**							
**Head and neck (** *n* ** = 37)**							
Nasopharynx Waldeyer’s ring CNS Orbit Sinus Parotid		10 11 11 2 1 2					
**Gastrointestinal tract (** *n* ** = 17)**							
Stomach Jejunum Ileum Colon		8 1 7 1					
Testis		1					
Bone		5					
Spleen		5					
Skin		4					
Liver		2					
Kidney and adrenal		1					
Bone marrow		4					
Breast		1					
**Subtype of NHL**		**Stage I** (*n* = 12)	**Stage II** (*n* = 65)		**Stage III** (*n* = 57)	**Stage IV** (*n* = 80)	
Diffuse Large B cell Lymphoma, including high-grade (*n* = 121)		6	38		33	40	
Follicular Lymphoma (*n* = 27)		1	6		4	16	
Mantle Cell Lymphoma (*n* = 8)		1	0		2	5	
Marginal Zone Lymphoma (*n* = 9)		4	4		0	1	
Peripheral T-Cell Lymphoma-NOS (*n* = 20)		0	7		5	8	
Anaplastic Large Cell Lymphoma (*n* = 5)		0	0		0	5	
Others		0	10		13	2	
**Prognostic Score**:							
**R-IPI** (*n* = 121)	**Frequency (n)**			**Percentage (%)**			
Very good	11/117			9.4%			
Good	47/117			40.17%			
Poor	57/117			48.72%			
Not Assigned	6/117			5.13%			
**FLIPI 1/2** (*n* = 27)	**Frequency (n)**			**Percentage (%)**			
Good	09/27			33.33%			
Intermediate	07/27			25.93%			
Poor	11/27			40.74%			
**MIPI** (*n* = 8)	**Frequency (n)**			**Percentage (%)**			
Low	2/8			25%			
Intermediate	2/8			25%			
High	2/8			25%			
Not Assigned	2/8			25%			

**Footnote: R-IPI**: Revised international prognostic index; **FLIPI**: follicular lymphoma international prognostic index; **MIPI**: mantle cell lymphoma international prognostic index

### Treatment

The majority of DLBCL received R-CHOP chemotherapy. Patients who were fit with good performance status, no significant cardiac comorbidities and aggressive disease such as double expressor, double hit, triple expressor, triple hit, plasmablastic lymphoma, TCR-BCL and PMBCL had received dose adjusted EPOCH-R. Low-grade lymphomas such as follicular, mantle cell and marginal zone lymphoma received B-R or R-CHOP and single-agent rituximab, respectively. Patients with PCNSL received R-MPV + radiotherapy + Ara-C whereas PTCL, ALCL and AITL were treated with CHOEP regimen. One case of PTCL and one case of relapsed refractory follicular lymphoma underwent autologous SCT with carmustine, etoposide, cytarabine and melphalan based conditioning chemotherapy with disease free 3 year survival. Only three cases of autologous stem cell transplant (1 case of relapsed hodgkins lymphoma and 2 cases were NHL) were done in our centre during the period between 2019 and 2022 due to covid 19 pandemic. Extra-nodal NK T Cell lymphoma was treated with dexamethasone, methotrexate, ifosphamide, l-asparaginase and etoposide (SMILE) regimen, but unfortunately, both patients succumbed to the disease.

### Safety

Grade 2 chemotherapy-related toxicity was noted in 116/214 patients (54.20%), of which the most common were gastrointestinal adverse effects such as nausea, vomiting, and mucositis, followed by grade 2–3 haematological toxicity and grade 2–3 hyponatremia which was managed conservatively. Table 2 enumerates the response rates to frontline treatment and Table 3 illustrates the various toxicities encountered. Grade 3 or more hepatobiliary, renal, electrolyte/glucose abnormalities, neurotoxicity, infusion site extravasation/infusion/injection-related/allergic reactions and anaphylaxis, and cytokine release syndrome were rarely encountered. Grade 1–2 toxicities were under-reported in various subgroups.

**Table 2:** Response to first-line therapy

	CR (*n* = 137)	PR (*n* = 20)	Stable (*n* = 10)	Progression (*n* = 43)
DLBCL including high grade^a^ (*n* = 121)	90	6	4	21
Follicular Lymphoma (*n* = 27)	17	3	2	4
Mantle Cell Lymphoma (*n* = 8)	5	1	0	0
Marginal Zone Lymphoma (*n* = 9)	9	0	0	0
PCNSL (*n* = 8)	4	1	1	1
PMBCL (*n* = 4)	2	1	0	1
Peripheral T-cell lymphoma (*n* = 19)	7	3	2	7
Anaplastic Large Cell Lymphoma (*n* = 4)	0	2	0	2
AITL (*n* = 2)	0	0	0	2
Other subtype^b^	3	3	2	5

**Footnote**: a. High-grade lymphoma include double expressor/triple expressor and double/triple hit

b. Include plasmablastic lymphoma, Waldenstrom’s macroglobulinemia, Burkitt’s lymphoma, T-cell rich B-cell lymphoma, extra-nodal NK-T cell lymphoma and hepatosplenic gamma-delta T-cell lymphoma; CR-complete response; PR-Partial response

**Table 3:** Regimen-related toxicity

Regimen related toxicity	*R*-CHOP (*n* = 97)	DA EPOCH-R (*n* = 14)	*R*-CODOX M / *R*-IVAC (*n* = 1)	CHOEP (*n* = 27)	R-MVP (*n* = 8)	B-R (*n* = 19)
Mucositis (*n* = 12)	0	9	1	0	2	0
Nausea/vomiting (*n* = 13)	0	6	1	2	4	0
Hyponatremia (*n* = 21)	11	4	0	4	2	0
Hepatic and renal dysfunction (*n* = 17)	12	2	1	1	0	1
Hematological toxicity (*n* = 21)	4	6	1	5	2	3
Vincristine neuropathy (*n* = 7)	3	2	0	2	0	0
Neutropenic Sepsis (*n* = 14)	0	7	1	2	4	0
Steroid myopathy (*n* = 9)	6	2	0	1	0	0
Cardiac toxicity (*n* = 2)	2	0	0	0	0	0

**Footnote: R-CHOP**: Rituximab Cyclophosphamide, Doxorubicin, Vincristine, Prednisolone; **DA-EPOCH R**: Dose adjusted Etoposide, Cyclophosphamide, Doxorubicin, Vincristine, Prednisolone, Rituximab; **R-CODOX M / R-IVAC**: Rituximab, Cyclophosphamide, Vincristine, Doxorubicin, Methotrexate/ Rituximab, Ifosphamide, Etoposide, Cytarabine; **CHOEP**: Cyclophosphamide, Doxorubicin, Vincristine, Etoposide, Prednisolone; **B-R**: Bendamustine, Rituximab; **R-MVP**: Rituximab, Methotrexate, Vincristine, Procarbazine

### Efficacy

After completion of treatment, the overall response rate was 73.36% (157/210). Response status was not captured in four patients due to loss of follow-up. Complete response was seen in 134/210 (63.2%), partial response in 23/210 (10.3%), and stable and progressive disease were seen in 10 and 43 patients, respectively, as described in Table 2. TCL and high-grade B NHL showed early relapse in 28 (14%) patients within 5 to 13 months of treatment completion. Eighteen patients died during chemotherapy due to disease progression, neutropenic sepsis and superior vena cava syndrome. On bivariate analysis, response to treatment was significantly associated with high-grade/low-grade B cell lymphoma and T-NHL (*p* = 0.002), stage of disease (*p* = 0.041) and IPI score (*p* = 0.002) as shown in Table 4. Other standard prognostic indicators such as bulky disease, performance status, LDH, beta-2-microglobulin, albumin, white cell count and haemoglobin were not evaluable for predicting response or outcome status due to inconsistent/missing data across subgroups.

**Table 4:** Analysis of disease parameters by response status

Characteristic	Total *N* = 214	Response status		*p*-value
		complete/partial response *N* = 157	stable/progression *N* = 53	
**Age**	53.3 ± 14.5	53.7 ± 14.0	52.0 ± 16.1	0.46
**Gender**				0.97
Male	159 (74.3%)	116 (73.9%)	39 (73.6%)	
Female	55 (25.7%)	41 (26.1%)	14 (26.4%)	
**Diagnosis**				0.002
High-grade B-cell lymphoma	135 (63.1%)	100 (63.7%)	31 (58.5%)	
Low-grade B-cell lymphoma	52 (24.3%)	44 (28.0%)	8 (15.1%)	
T-cell lymphoma	27 (12.6%)	13 (8.3%)	14 (26.4%)	
**Stage of disease**				0.041
Stage I and II	77 (36.0%)	63 (40.1%)	13 (24.5%)	
Stage III and IV	137 (64.0%)	94 (59.9%)	40 (75.5%)	
**IPI**				0.002
Good / Low	69 (46.6%)	62 (53.0%)	6 (20.7%)	
Poor / Intermediate / High	79 (53.4%)	55 (47.0%)	23 (79.3%)	
**Outcome**				< 0.001
Alive	106 (49.53%)	97 (61.8%)	7 (13.2%)	
Death / Relapse / Progression	108 (50.46%)	60 (38.2%)	46 (86.8%)	

Note: Response status unavailable in four patients due to loss of follow-up

### Outcome

**Fig. 2:**
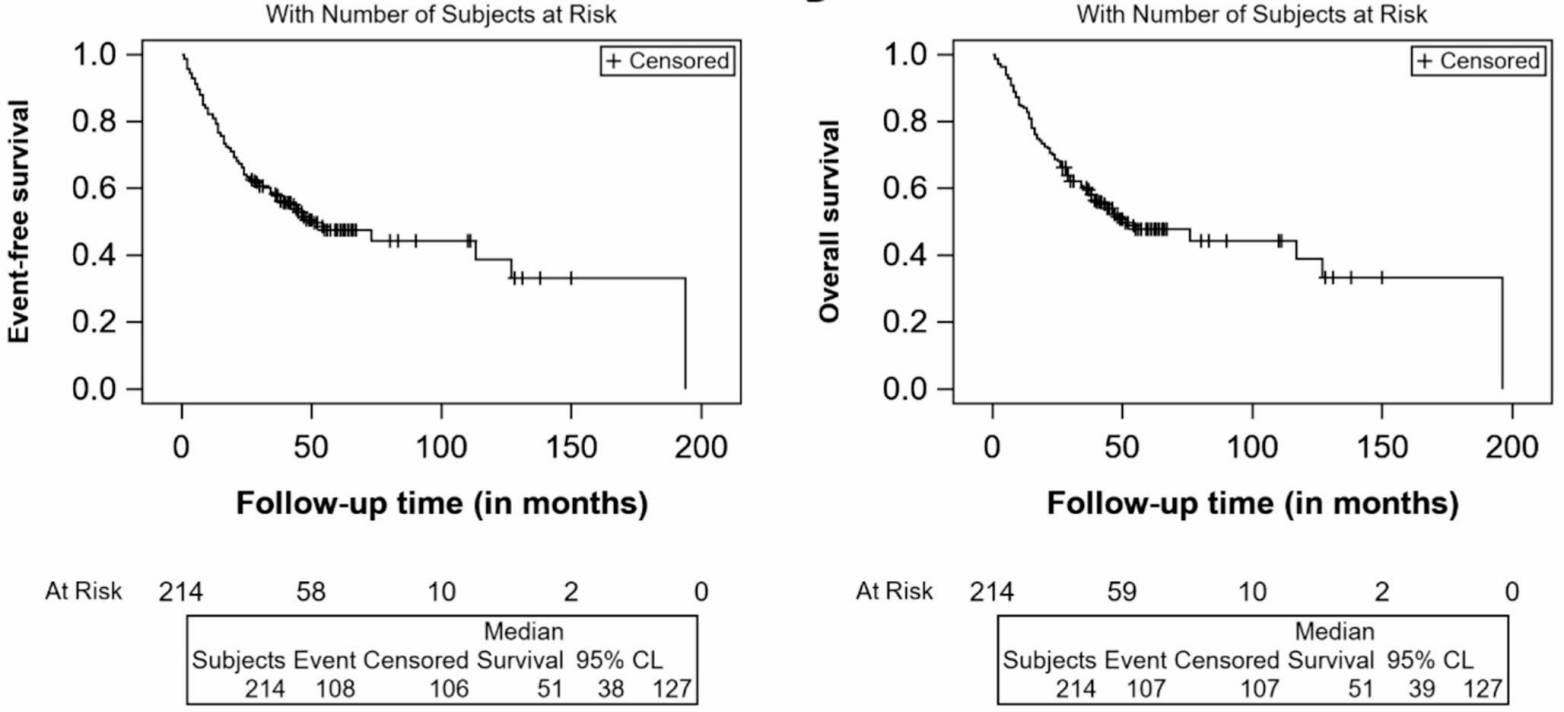
Kaplan-Meier survival curves showing event-free and overall survival of the entire cohort

**Fig. 3:**
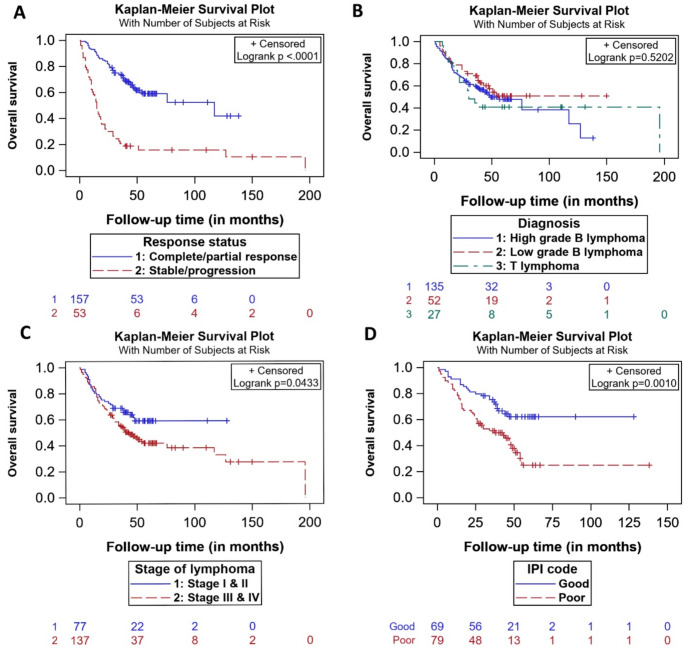
Kaplan-Meier survival curves depicting overall survival by (**a**) response status, (**b**) diagnosis, (**c**) stage of lymphoma and (**d**) IPI code. Kaplan Meier curves for overall survival for (**A**) complete and partial treatment response (blue), stable/progressive disease (red). (**B**) high-grade B lymphoma (blue), low-grade B lymphoma (red), and T lymphoma (green). (**C**) stage I and II (blue), stage III and IV(red). (**D**) good/low-risk IPI group (blue), poor/intermediate/high IPI risk (red).

The 2-year event-free and overall survival for the entire cohort was 64.02% (95% CI, 57.02–70.04%) and 68.69% (95% CI, 62.01–74.44%). Figure 2 depicts the event-free and overall survival of the entire cohort. Those who responded to first-line therapy had 2-year EFS and OS of 77.7% (95% CI, 70.36–83.44%) and 82.17% (75.23–87.32%) compared to 24.53% (95% CI, 13.99–36.63%) and 30.19% (18.53–42.7%)in those who did not respond to treatment. The 5-year OS of all patients with NHL was 47.84% (95% CI, 40.23–55.03%), and those who had good /low-risk IPI had 5-year OS of 62.09% (95% CI, 48.47–73.07%) than those with high/poor risk IPI of 25.17% (95% CI, 12.15–40.53%). Most events occurred within 6 to 24 months; nearly 72 out of 108 events (66.66%) happened within 24 months. At the date of the last follow-up, 108 developed events, of which 67 had a relapse, 14 had progression, and 106/214 were alive without relapse or progression. The overall survival by (a) response status, (b) diagnosis, (c) stage of lymphoma and (d) IPI code are depicted in Fig. 3 below.

### Follow-up

The median follow-up of patients was 23 months (IQR 15–35 months). Response status was not captured in four patients due to loss of follow-up. Out of the remaining 210 patients, 106 developed events or died during follow-up. Out of 53 patients who experienced stable/progressive disease, 46 (86.8%) died, with a median survival of 1 year 3 months (95% CI 10–19 months). Of 157 patients who had a partial or complete response, 59 (37.6%) died, with a median survival of 9 years and 9 months. Among patients with high-grade, low-grade B NHL and TCL, median follow-up time was 38 months (IQR,10–51 months), 40.5 months(IQR,25–56 months) and 30 months(IQR,19–51 months), respectively. Similarly, the median follow-up for limited and advanced stages was 40 months (IQR, 22–55 months) and 38 months (IQR, 16–51 months), respectively. We found a significant difference (*p* = 0.002) in median follow-up between low-risk IPI (42. months; IQR, 34 to57 months) and high-risk (30 months; IQR, 15 to 45 months), but there was no significant difference in the median follow-up times in patients with limited /advanced disease (*p* = 0.50) as well as between high grade/low grade or TCL.(*p* = 0.38). There was also a significant difference in median follow-up times of patients who achieved complete/partial response (43 months; IQR, 30–56 months) than those who did not respond (15 months, IQR,7–30 months).

### Cox Regression Results

Though advanced stage (HR = 1.55; 95%CI, 1.01 to 2.37), poor IPI (HR = 2.23; 95% CI, 1.36 to 3.66) and inferior treatment response (HR = 4.21; 95% CI, 2.83 to 6.27) predicted lower overall survival only response to treatment and IPI score predicted EFS in univariate (unadjusted) analysis. In particular, poor IPI score (HR 2.12; 95% CI 1.30–3.45) and stable/progressive disease (HR 4.20; 95% CI 2.82–6.25) had high risk of event or death. On multivariable (adjusted) analysis, response to treatment, whether stable/progressive disease (HR 5.06; 95% CI 2.98–8.61), was the only independent predictor of EFS and OS, as shown in Table 5.

**Table 5:** Cox multiple regression results for risk factors associated with overall survival and event-free survival:

	Overall survival (OS)				Event-free survival (EFS)			
	Univariate analysis(unadjusted model)		Multivariate analysis(adjusted model)		Univariate analysis(unadjusted model)		Multivariate analysis(adjusted model)	
	HR (95% CI)	*p*-value	HR (95%CI)	*p*-value	HR (95%CI)	*p*-value	HR (95%CI)	*p*-value
**Age**	1.01 (0.99,1.02)	0.422	-	-	1 (0.99,1.02)	0.562	-	-
**Gender** (Referrent: Male) Female	1.2 (0.78,1.86)	0.4	-	-	1.18 (0.76,1.81)	0.462	-	-
**Diagnosis** (Referrent: High-grade B-cell lymphoma) Low-grade B-cell lymphoma	0.79 (0.49,1.27)	0.329	-	-	0.78 (0.49,1.25)	0.304	-	-
T-cell lymphoma	1.1 (0.63,1.91)	0.734	-	-	1.1 (0.63,1.90)	0.744	-	-
**Stage** (Referrent: stage I and II) Stage III and IV	1.55 (1.01,2.37)	0.046	1.8 (0.90,3.60)	0.095	1.49 (0.98,2.28)	0.064	1.72 (0.88,3.37)	0.113
**IPI** (Referrent: Low/Good) High/Poor IPI	2.23 (1.36,3.66)	0.001	1.38 (0.78,2.43)	0.268	2.12 (1.30,3.45)	0.003	1.33 (0.76,2.32)	0.325
**Response** (Referrent: Complete/partial response) Stable/progression	4.21 (2.83,6.27)	< 0.001	5.07 (2.98,8.61)	< 0.001	4.20 (2.82,6.25);	< 0.001	5.06 (2.98,8.61)	< 0.001

## Discussion

There was an increasing incidence of NHL compared to a previous study conducted at the same centre ten years back by Hazarika et al. [10]. There was a male predominance of 74.7%, and age over 45 constituted 68.2%. Cervical lymphadenopathy was the most typical presentation. The presentation of bulky disease was 12.1%, and extra-nodal disease was 35.98%, similar to data from Delhi [11].Head and neck (48%), followed by gastrointestinal tract, were common sites, comparable to other studies [12, 13].Isolated kidney, adrenal, breast, and testicular involvement were rarely noted. Diffuse large B-cell, follicular lymphoma and PTCL were common, as reported in various literature [14–16].More than half belonged to the advanced stage due to inadequate access to quality health care, delayed referral and fewer oncology centres. Only 14.4% had very good/low IPI; the rest were intermediate, high or poor risk. Though DLBCL was commonest, double or triple expressor phenotype, bulky disease, advanced stage, extra-nodal presentation, poor IPI attributed to lower response and poor outcome even in young age group. Rare subtypes such as PMBCL, plasmablastic lymphoma, AITL, NK-TCL and hepatosplenic gamma delta TCL had worse response rates and dismal outcomes despite standard regimens in our study as well in other data [17, 18]. T-cell lymphoma showed poor response to conventional regimens across all age groups due to limited access to autologous transplants, necessitating novel options to enhance survival. Advanced stage, high/high-intermediate score by Prognostic index for PTCL unspecified (PIT) for PTCL, lack of consolidative stem cell transplant accounted for early relapse, inferior response and dismal 5-year outcome in T-NHL.

Grade II anaemia, neutropenia, thrombocytopenia, and hyponatremia were frequently encountered toxicities, followed by hepatic/renal dysfunction and neutropenic sepsis due to a high proportion of high-grade lymphomas such as double expressor or double hit, which were comparable to other Indian data but higher than western literature [19–21].No death occurred due to chemotherapy toxicity. Use of supportive care in the form of prolonged hospital stay, blood product transfusion, antibiotics and G-CSF were less and mainly advised in the elderly, those with poor performance status, comorbidities and high-grade disease. The overall response rate was 73.36% with a complete response of 63.2% among whole cohort. This is slightly higher than previous data published from our institute [10], however, lower than developed countries due to advanced presentation [22–24]. As a standard prognostic factor and surrogate disease indicator, baseline IPI predicted the response rate to first-line treatment and outcome across aggressive and significant subtypes.

Limited stage, lower baseline IPI of 0–2 and response to 1st line had better overall survival (68.69% at 2 years; 47.84% at 5 years, and event-free survival of 64.02% at 2 years; 47.64% at 5 years) compared to stage III/IV, IPI of 3–6 or inadequate treatment response. This was slightly higher than previous data published by Prakash et al. in 2012 due to the precise sub-classification of NHL as per the 2016 WHO classification, where the cell of origin based on the Hans algorithm to identify GCB and non-GCB subtype and consistent molecular markers to identify high-grade BCL has promptly enhanced outcome in HGBCL over last decade [25]. OS and 2-year EFS were similar to few Indian data but were slightly less than OncoCollect lymphoma registry data published by Nair et al. and study on cell of origin by Gogia et al. which primarily comprised DLBCL [26, 27]. The possible explanation for lower survival in our data could be due to heterogenous analysis of NHL incorporating high grade DLBCL such as double expressor, burkitts lymphoma, PMBCL, hepatosplenic gamma delta TCL and NK-TCL and lack of consolidative SCT.

Most adhered to treatment protocol despite advanced, bulky and high-grade disease biology and overall survival at 2 years and 5 years were non-inferior to previous studies in India [25–28].Achieving complete/partial response to frontline immunochemotherapy was significantly associated with better 2-year EFS and OS compared to stable/progressive disease. Table 6 compares the current study with various studies in India and Western data. Poor/high IPI score, advanced stage and suboptimal response to frontline therapy were significantly associated with worse 5-year OS in our study. As well studied from previous literature, our data also showed the occurrence of maximum events during the first 6–18 months of follow-up and, after that, a decline in the probability of events and few relapses. The likelihood of relapse, disease progression or death declined proportionately after 24 months. We noted around 38 events, including relapses and death at 12 months of treatment completion, followed by 72 deaths or relapses at the end of 24 months, similar to other Indian data [25–28].Similarly, though the median follow-up was 23 months, there was a significantly lower median follow-up among poor/high IPI and inadequate response groups. Hence, suboptimal responses to frontline therapy had inferior overall survival than those who responded.

**Table 6:** Comparison of the present study with Western and Indian data:

	Current study	US SEER data [3], All NHL	Li et al., 2020, [29], All high-grade B-cell NHL,	Prakash et al., 2012, [25], all NHL, survival for DLBCL	Gogia et al., 2021, [27] only DLBCL	Karunakaran et al., 2022, [28] all high grade B- NHL	Nair et al., 2022 [26], All DLBCL	Same centre, 2015, [10] All NHL	Fetica et al., 2017 [30]
**Total patients**,** number**	214	61,214	41	260	417	30	1961	111	184
**Median age**,** in years**	54	79% were more than 50	26.8% were > 60 years	50	48	49.3	57	54	57
**Male/Female ratio**	2.8:1		63.4% males	2.2:1	2:1	73.3% males	1.6:1	1.9:1	0.94
**Limited stage**	36%	44	-	46%	-	46.7%	43%	55%	-
**Advanced stage**	64.%	56	56.1%	54%	51%	53.3%	57%	45%	-
**Extra-nodal presentation**	35.98%	21	56.1%, >1 extra-nodal region	31%	-	60%	53%	63.1% (nodal + extra-nodal )	-
**Bulky disease**	12.1%	-	-	35%	35.5%	40%	10.33%	-	-
**High/Poor IPI**	53.4%	-	58.5%	-	61.1%	66.6%, (NCCN-IPI > 2)	29.92%	35.1%	-
**No response to 1st line**	24.76%	-	41.4%	-	15%	26.6%	12.8%	52.25%	-
**EFS**	64.02% at 2 years; 47.64% at 5 years	Not reached	-	54%	74.7% (3-year EFS for GCB)	22 months	66.58%	-	-
**OS**	68.69% at 2 years;47.84% at 5 years	50% at 5 years	36.7% at 2 years	64.7%	88% (3 year OS)	68% at years	75.37%	-	41.7% at 5 years for TCL; 32.9% for DLBCL

### Limitations

Being a single-centre experience, selection bias, small subgroup analysis capping significance among observations, various missing data contributing to the evaluation of salient disease parameters, unequal distribution of prognostic factors, geographic variations in phenotype and modifiers of treatment result challenge the comparison across various retrospective studies. Further, the data on the distribution of patient characteristics among high-grade/low-grade B NHL and IPI were not analogous. Given the COVID-19 pandemic, variability in treatment decisions, staging, response and follow-up evaluation limit the generalizability of findings. In comparison with prospective and randomized controlled studies, caution is to be exercised when interpreting OS and EFS as loss to follow up, details on first or multiple relapses, second or third line treatment selection, response, and outcome after various lines of treatment were not captured reflecting the need for comprehensive data collection platform in high volume referral centres.

## Conclusion

Despite being a resource-limited setting encompassing practical challenges and limitations, the overall response rate was non-inferior and comparable to Western data. High-grade disease, T-NHL, advanced stage posed significant constraints in achieving optimal survival, thus emphasizing the incorporation of novel prognostic markers and biological agents based upon cell of origin, high-risk molecular signature, and epigenetic alterations, enabling individualized therapy to limit relapse and progression in this subset. Pitfalls of retrospective design highlight the need for extensive multicenter studies and randomized controlled trials evaluating molecular subgroups based on gene-expression profiling, targeted agents and CAR-T therapy, are recommended as future prospects.

## Data Availability

All data were collected from hospital electronic medical records (EMR) and patient case files.
